# Different N—H⋯π inter­actions in two indole derivatives

**DOI:** 10.1107/S2056989016006162

**Published:** 2016-04-15

**Authors:** Jamie R. Kerr, Laurent Trembleau, John M. D. Storey, James L. Wardell, William T. A. Harrison

**Affiliations:** a Department of Chemistry, University of Aberdeen, Meston Walk, Aberdeen AB24 3UE, Scotland; b Fundação Oswaldo Cruz, Instituto de Tecnologia em Fármacos-Far Manguinhos, 21041-250 Rio de Janeiro, RJ, Brazil

**Keywords:** crystal structure, indole, N—H⋯π inter­action, chains, inversion dimers

## Abstract

We describe the syntheses and crystal structures of two indole derivatives, namely 6-isopropyl-3-(2-nitro-1-phenyl­eth­yl)-1*H*-indole, C_19_H_20_N_2_O_2_, (I), and 2-(4-meth­oxy­phen­yl)-3-(2-nitro-1-phenyl­eth­yl)-1*H*-indole, C_23_H_20_N_2_O_3_, (II); the latter crystallizes with two mol­ecules (*A* and *B*) with similar conformations (r.m.s. overlay fit = 0.139 Å) in the asymmetric unit. Despite the presence of O atoms as potential acceptors for classical hydrogen bonds, the dominant inter­molecular inter­action in each crystal is an N—H⋯π bond, which generates chains in (I) and *A*+*A* and *B*+*B* inversion dimers in (II). A different aromatic ring acts as the acceptor in each case. The packing is consolidated by C—H⋯π inter­actions in each case but aromatic π–π stacking inter­actions are absent.

## Abstract 2

The most important inter­molecular inter­actions in the two indole derivatives described here are N—H⋯π bonds, which lead to chains in one case and inversion dimers in the other; C—H⋯π inter­actions appear to reinforce the N—H⋯π bonds in each case.

## Chemical context   

N—H⋯π inter­actions are now a well-recognised type of ‘non-classical’ weak bond (Desiraju & Steiner, 1999 ▸). They are of special significance in biological systems (Burley & Petsko, 1986 ▸; Levitt & Perutz, 1998 ▸) and are thought to play an important role in establishing protein secondary structures (Lavanya *et al.*, 2014 ▸). They may even influence the charge-transport properties of organic semiconductors (Zhao *et al.*, 2009 ▸). The presence of N—H⋯π inter­actions in indole complexes with aromatic species has been investigated by IR spectroscopy (Muñoz *et al.*, 2004 ▸), and such bonds have also been observed in many crystal structures of indole derivatives (*e.g*. Krishna *et al.*, 1999 ▸; Cordes *et al.*, 2011 ▸).
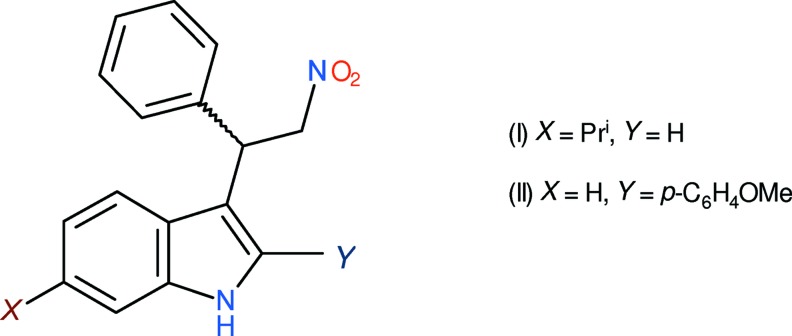


As part of our ongoing synthetic, biological (Kerr, 2013 ▸) and structural studies (Kerr *et al.*, 2015 ▸, 2016 ▸) of variously substituted indole derivatives, we now report the syntheses and crystal structures of 6-isopropyl-3-(2-nitro-1-phenyl­eth­yl)-1*H*-indole, C_19_H_20_N_2_O_2_, (I), and 2-(4-meth­oxy­phen­yl)-3-(2-nitro-1-phenyl­eth­yl)-1*H*-indole, C_23_H_20_N_2_O_3_, (II), in which N—H⋯π bonds are the most important inter­molecular inter­actions, but result in quite different structures.

## Structural commentary   

**Figure 1:**
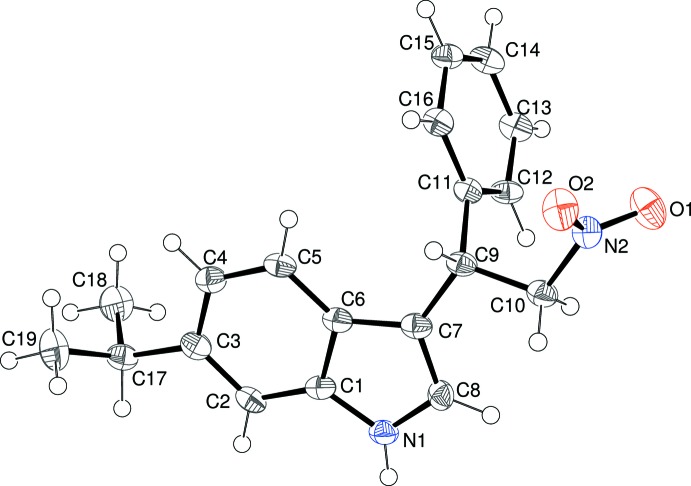
The mol­ecular structure of (I), showing 50% probability displacement ellipsoids.

Compound (I) crystallizes in a Sohncke space group with one mol­ecule in the asymmetric unit (Fig. 1 ▸). The absolute structure was indeterminate in the present study and C9 was assigned an arbitrary *S* configuration (given the synthesis, we presume that the bulk sample consists of a statistical mixture of enanti­omers). The dihedral angle between the mean plane of the N1/C1–C8 indole ring system (r.m.s. deviation = 0.018 Å) and the C11–C16 phenyl ring is 83.59 (11)°. Atom C17 of the 6-isopropyl substituent deviates slightly from the indole plane, by −0.092 (6) Å. In terms of the terminal carbon atoms of this group, C18 and C19 deviate from the indole plane by −1.461 (6) and 1.030 (6) Å, respectively. Atom C9 shows a relatively large deviation from the indole plane of −0.084 (6) Å, perhaps because of steric crowding. In terms of the orientation of the substituents attached to C9, the C6—C7—C9—C10 torsion angle of 174.6 (5)° (*anti* about C7—C9) indicates that the C10 atom of the CH_2_NO_2_ group lies roughly in the plane of the indole ring, whereas the C6—C7—C9—C11 angle of −61.6 (7)° (*gauche* about C7—C9) indicates that the pendant ring lies to one side of the indole plane. Finally, the C7—C9—C10—N2 torsion angle of −176.5 (4)° indicates a near *anti* conformation about the C9—C10 bond.

**Figure 2:**
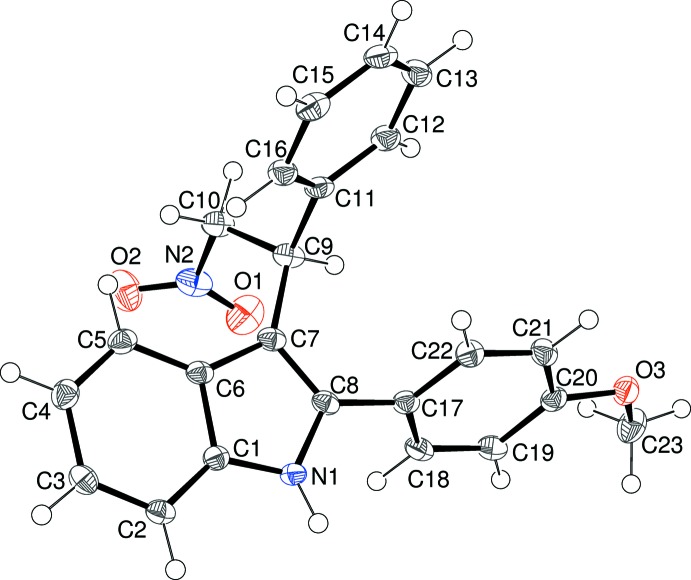
The mol­ecular structure of the N1 mol­ecule in (II), showing 50% probability displacement ellipsoids. The mol­ecular structure of the N3 mol­ecule is very similar.

There are two mol­ecules, *A* (Fig. 2 ▸) and *B*, in the asymmetric unit of (II). The space group for (II) is centrosymmetric and the stereogenic centres (C9 in mol­ecule *A* and C32 in mol­ecule *B*) were arbitrarily assigned an *S* configuration for ease of comparison with compound (I).

**Figure 3:**
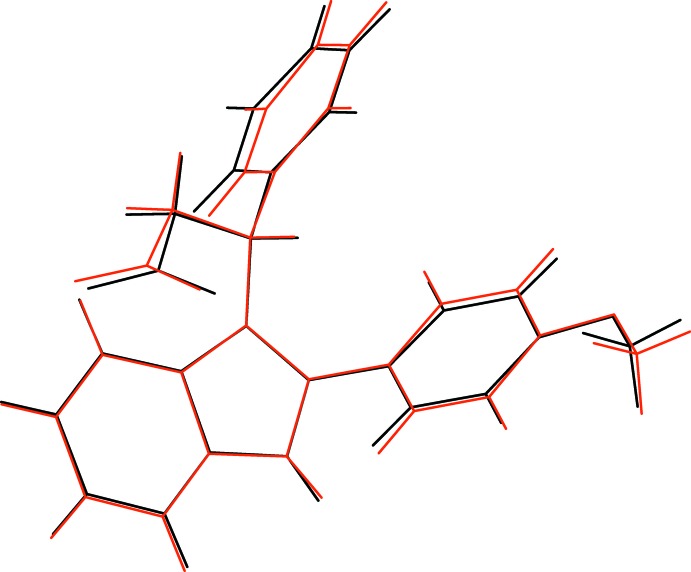
Overlay plot of the conformations of the N1 mol­ecules (black) and N3 mol­ecules (red) in the crystal of (II).

In mol­ecule *A*, the dihedral angles between the indole (N1/C1–C8) mean plane (r.m.s. deviation = 0.012 Å) and the C11–C16 and C17–C22 rings are 65.49 (4) and 66.26 (4)°, respectively. The deviations of C9 and C17 from the indole plane are 0.017 (2) and 0.0168 (19) Å, respectively; C23 deviates from the C17–C22 plane by 0.322 (3) Å. The equivalent data for mol­ecule *B* are 0.005 Å (N3/C24–C31 r.m.s. deviation), 64.92 (4)° (C34 ring), 58.31 (5)° (C40 ring), −0.071 (2) Å (C32), −0.014 (2) Å (C40), −0.214 (3) Å (C46). These data indicate that mol­ecules *A* and *B* have similar but not quite identical conformations: the unweighted r.m.s. overlay fit for the 28 non-hydrogen atoms is 0.139 Å (Fig. 3 ▸).

As just noted, mol­ecules *A* and *B* in (II) have similar conformations, but the local geometry about the stereogenic atoms C9 and C32 are completely different from the corresponding local geometry about C9 in (I). This can be seen in the following data for the N1 mol­ecule in (II): the C6—C7—C9—C10 torsion angle is −42.9 (2)° (compressed *gauche* about C7—C9) and the C6—C7—C9—C11 angle is 83.76 (19)° (expanded *gauche* about C7—C9); the C7—C9—C10—N2 torsion angle of −58.42 (17)° (*gauche* about C9—C10) is also completely different from the corresponding angle in (I). The corresponding torsion angles for the N3 mol­ecule in (II) are −38.4 (2), 87.50 (19) and −56.24 (19)°, respectively. In essence, the 2-nitro 1-phenyl ethyl substituent has rotated around the C7—C9 bond, so that the H atom attached to C9 and C32 in (II) lies approximately above C8 whereas in (I) the CH_2_NO_2_ group takes on this role.

## Supra­molecular features   

**Figure 4:**
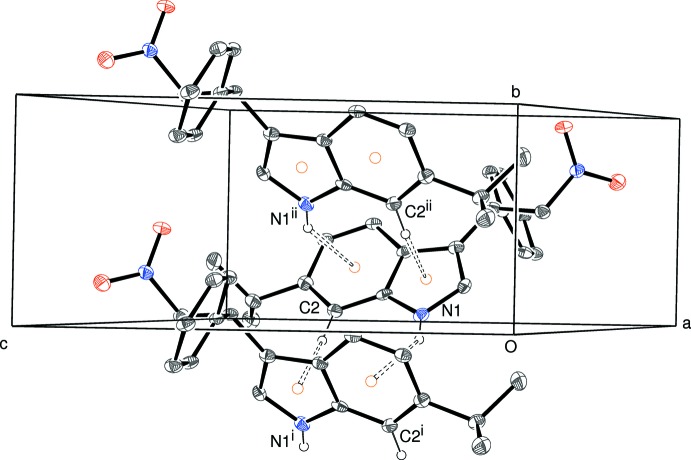
Partial packing diagram for (I), showing the formation of [010] chains linked by N—H⋯π and C—H⋯π inter­actions (double-dashed lines). [Symmetry codes: (i) 1 − *x*, *y* − 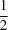, 1 − *z*; (ii) 1 − *x*, *y* + 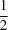, 1 − *z*.] All H atoms, except H1 and H2, have been omitted for clarity. The orange circles indicate ring centroids.

**Figure 5:**
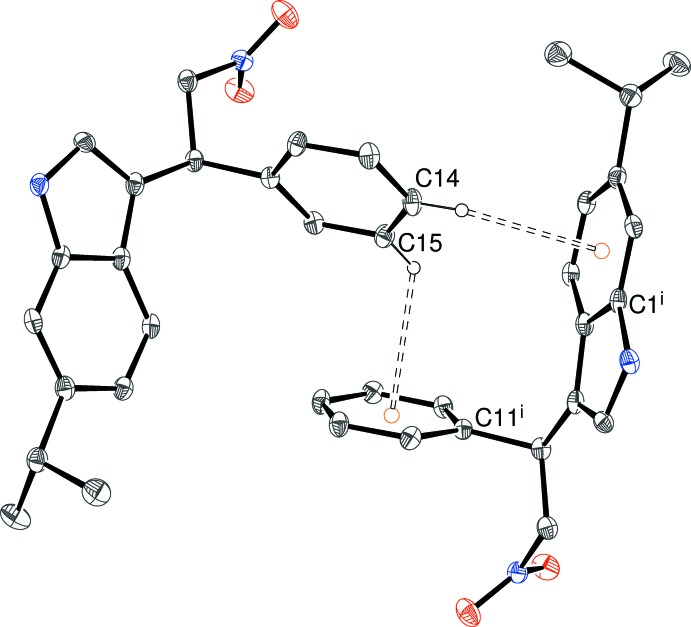
Fragment of the packing for (I), showing C—H⋯π bonds arising from adjacent C—H groups of the pendant benzene ring. All H atoms, except H14 and H15, have been omitted for clarity. [Symmetry code: (i) 1 − *x*, *y* + 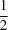, −*z*.] The orange circles indicate ring centroids.

In the crystal of (I), the mol­ecules are linked by N—H⋯π inter­actions (Table 1 ▸, Fig. 4 ▸) to generate [010] chains, in which adjacent mol­ecules are related by the 2_1_ screw axis. The acceptor ring is the C1–C6 benzene ring of the indole system; the dihedral angle between any adjacent pair of indole ring systems in the chain is 68.89 (8)°. The chain appears to be reinforced by a C—H⋯π bond from the C2—H2 group of the benzene ring *syn* to the N—H group to the five-membered ring of the same adjacent mol­ecule; the H⋯π separation is actually marginally shorter for this bond than for the N—H⋯π bond. Two further C—H⋯π inter­actions (Fig. 5 ▸) also occur in the crystal of (I): based on their lengths, these are presumably significantly weaker than the C2—H2 bond. They arise from adjacent C—H groups on the pendant C11–C16 benzene ring with the acceptor rings being another C11–C16 ring and the C1–C6 indole ring of the same adjacent mol­ecule. Taken together, the inter­molecular inter­actions lead to (100) sheets in the crystal of (I).

**Figure 6:**
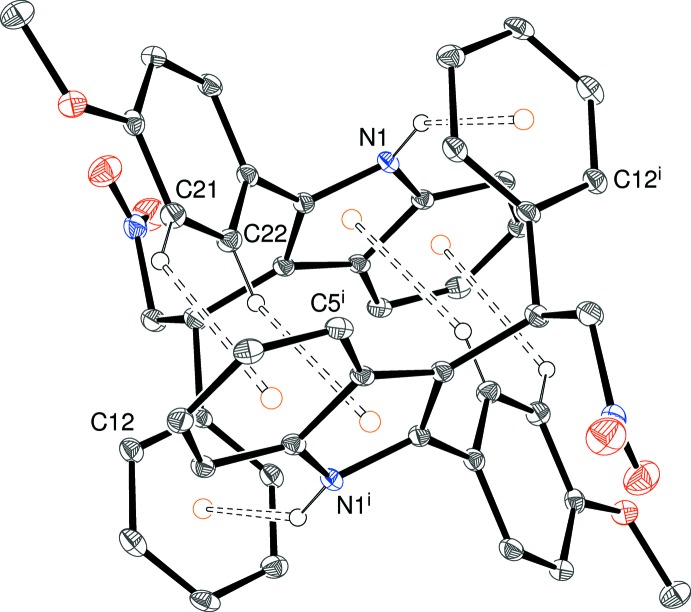
An inversion dimer of N1 mol­ecules in the crystal of (II) linked by pairs of N—H⋯π and C—H⋯π inter­actions (double-dashed lines). [Symmetry code: (i) 1 − *x*, −*y*, −*z*.] The N3 mol­ecules associate into similar dimers. The orange circles indicate ring centroids.

In the crystal of (II), inversion dimers linked by pairs of N—H⋯π inter­actions (Table 2 ▸, Fig. 6 ▸) occur for both independent mol­ecules. In this case, the acceptor ring is the pendant C11–C16 or C34–C39 benzene ring for mol­ecules *A* and *B*, respectively. This bonding mode possibly correlates with the different orientation of the substituents attached to C9 and C32, as described above. Again, the N—H⋯π bonds appear to be reinforced, but this time by *two* pairs of C—H⋯π inter­actions. As for (I), they arise from adjacent C—H groups in a benzene ring but this time they are part of the pendant 4-meth­oxy­benzene ring at the indole 2-position. Further C—H⋯π bonds link the *A*+*A* and *B*+*B* dimers into a three-dimensional network in the crystal of (II).

## Database survey   

There are over 7000 crystal structures of indole derivatives in the Cambridge Structural Database (CSD; Groom *et al.*, 2016 ▸), but none of them have an iso-propyl group at the 6-position. Six structures contain a *p*-meth­oxy­benzene grouping at the 2-position and four contain a 2-nitro-1-phenyl­ethyl grouping at the 3-position; these latter structures are the ones recently described by us (Kerr *et al.*, 2015 ▸).

## Synthesis and crystallization   

To prepare (I), 6-iso­propyl­indole (452 mg, 2.84 mmol), *trans*-β-nitro­styrene (28, 429 mg, 2.88 mmol) and sulfamic acid (57 mg, 0.59 mmol) were stirred in EtOH (10 ml) at 323 K for 48 h. Evaporation of the solvent and flash chromatography (1:6 EtOAc, hexa­nes) gave 6-isopropyl-3-(2-nitro-1-phenyl­eth­yl)-1*H*-indole as an orange solid (550 mg, 63%). Red blades of (I) were recrystallized from methanol solution. δC (101 MHz; CDCl_3_) 144.0 (Cq), 139.3 (Cq), 136.9 (Cq), 127.9 (Cq), 127.8 (CH), 127.5 (CH), 124.3 (CH), 121.1 (CH), 119.4 (CH), 118.6 (CH), 114.3 (Cq), 108.5 (CH), 79.5 (CH_2_), 41.6 (CH), 34.3 (CH) and 24.4 (CH_3_); δH (400 MHz; CDCl_3_) 7.89 (1 H, *br s*), 7.30–7.21 (5 H, *m*), 7.18–7.15 (1 H, *m*), 7.12 (1 H, *t*, *J* 0.6), 6.90 (2 H, *td*, *J*, 1.5, 8.8), 5.08 (1 H, *t*, *J* 8.0), 4.97 (1 H, *dd*, *J* 7.4, 12.2), 4.85 (1 H, *dd*, *J* 8.4, 12.4), 2.91 (1 H, *sp*, *J* 6.9) and 1.20 (6 H, *d*, *J* 6.8); *R*_f_ 0.16 (1:6 ethyl acetate, hexa­nes); m.p. 374–376 K; IR (KBr, cm^−1^) 3433, 3007, 2924,1550, 1429, 1377, 1089 and 750; HRMS (ESI) for C_19_H_21_N_2_O_2_ [*M* + H]^+^ calculated 309.1604, found 309.1619.

To prepare (II), 2-bromo-3-(2-nitro-1-phenyl­eth­yl)-1*H*-indole (Kerr *et al.*, 2015 ▸) (90 mg, 0.26 mmol), 4-meth­oxy­phenyl­boronic acid (53 mg, 0.35 mmol), Na_2_CO_3_ (29 mg, 0.27 mmol), LiCl (22 mg, 0.52 mmol) and tetra­kis­(tri­phenyl­phosphine)palladium(0) (12 mg, 0.01 mmol) were placed in a microwave reactor vessel under argon. Degassed water (4 ml), toluene (6 ml) and ethanol (6 ml) were added and the reaction was heated to 373 K (high absorbance mode, 30 W, 8 bar) for 2 h. The mixture was acidified to pH 2 with 10% HCl(aq) then extracted into EtOAc (10 ml × 3). The combined organic phases were washed with water (10 ml) and saturated NaCl(aq) (10 ml) then dried (magnesium sulfate), filtered and evaporated under reduced pressure. Flash chromatography of the isolated solid (1:5 ethyl acetate, hexa­nes) afforded 2-(4-meth­oxy­phen­yl)-3-(2-nitro-1-phenyl­eth­yl)-1*H*-indole as a colourless solid (48 mg, 50%). Colourless chunks of (II) were recrystallized from methanol solution. δC (63 MHz; CDCl_3_) 159.9 (Cq), 140.0 (Cq), 136.9 (Cq), 135.9 (CH), 130.1 (CH), 128.9 (Cq), 127.1 (CH), 125.0 (CH), 124.5 (Cq), 122.2 (Cq), 120.2 (CH), 119.8 (CH), 114.4 (CH), 111.3 (CH), 110.0 (CH), 109.1 (Cq), 79.1 (CH_2_), 55.4 (CH_3_) and 40.9 (CH); δH (250 MHz; CDCl_3_) 8.08 (1 H, *br s*), 7.45–7.25 (9 H, *m*), 7.19–6.90 (4 H, *m*), 5.19 (1 H, *t*, *J* 6.9) 5.10–5.01 (2 H, *m*) and 3.76 (3 H, *s*); *R*_f_ 0.09 (1:5 EtOAc, hexa­nes); m.p. 472 K (EtOH); IR (Nujol, cm^−1^) 3401, 3013, 2854, 1616, 1548, 1324, 1250, 1203, 1099, 870 and 746; HRMS (ESI) for C_23_H_21_N_2_O_3_ [*M* + H]^+^ calculated 373.1553, found 373.1546.

## Refinement   

Crystal data, data collection and structure refinement details are summarized in Table 3 ▸. The N-bound H atoms were located in difference maps and their positions were freely refined. The C-bound H atoms were geometrically placed (C—H = 0.93–0.98 Å) and refined as riding atoms. The constraint *U*_iso_(H) = 1.2*U*_eq_(C, N carrier) or 1.5*U*_eq_(methyl carrier) was applied in all cases. The –CH_3_ groups were allowed to rotate, but not to tip, to best fit the electron density. Due to the similarity in the *a* and *c* unit-cell parameters for (I), twinning models were applied, but no improvement in fit resulted.

## Supplementary Material

Crystal structure: contains datablock(s) I, II, global. DOI: 10.1107/S2056989016006162/pk2578sup1.cif

Structure factors: contains datablock(s) I. DOI: 10.1107/S2056989016006162/pk2578Isup2.hkl

Structure factors: contains datablock(s) II. DOI: 10.1107/S2056989016006162/pk2578IIsup3.hkl

Supporting information file. DOI: 10.1107/S2056989016006162/pk2578Isup4.cml

Supporting information file. DOI: 10.1107/S2056989016006162/pk2578IIsup5.cml

CCDC references: 1473664, 1473663

Additional supporting information:  crystallographic information; 3D view; checkCIF report

## Figures and Tables

**Table 1:** Hydrogen-bond geometry (Å, °) for (I) *Cg*1, *Cg*2 and *Cg*3 are the centroids of the N1/C1/C6–C8, C1–C6 and C11–C16 rings, respectively.

*D*—H⋯*A*	*D*—H	H⋯*A*	*D*⋯*A*	*D*—H⋯*A*
N1—H1⋯*Cg*2^i^	0.84 (6)	2.64 (6)	3.386 (5)	148 (6)
C2—H2⋯*Cg*1^i^	0.95	2.63	3.468 (6)	147
C14—H14⋯*Cg*2^ii^	0.95	2.79	3.638 (6)	148
C15—H15⋯*Cg*3^ii^	0.95	2.87	3.551 (7)	129

Symmetry codes: (i) 
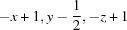
; (ii) 
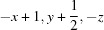
.

**Table 2:** Hydrogen-bond geometry (Å, °) for (II) *Cg*1, *Cg*2, *Cg*3, *Cg*6, *Cg*7 and *Cg*8 are the centroids of the N1/C1/C6–C8, C1–C6, C11–C16, N3/C24/C29–C34--C31, C24–C29 and C34–C39 rings, respectively.

*D*—H⋯*A*	*D*—H	H⋯*A*	*D*⋯*A*	*D*—H⋯*A*
N1—H1⋯*Cg*3^i^	0.886 (19)	2.640 (19)	3.3631 (15)	139.6 (15)
N3—H3⋯*Cg*8^ii^	0.875 (18)	2.582 (19)	3.3364 (15)	144.9 (16)
C14—H14⋯*Cg*2^iii^	0.95	2.58	3.4228 (18)	149
C21—H21⋯*Cg*2^i^	0.95	2.69	3.4133 (17)	134
C22—H22⋯*Cg*1^i^	0.95	2.68	3.4543 (17)	138
C23—H23*B*⋯*Cg*3^iv^	0.98	2.79	3.6739 (18)	150
C37—H37⋯*Cg*6^v^	0.95	2.86	3.7660 (18)	160
C41—H41⋯*Cg*6^ii^	0.95	2.70	3.3793 (17)	129
C42—H42⋯*Cg*7^ii^	0.95	2.67	3.3627 (17)	130

Symmetry codes: (i) 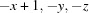; (ii) 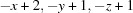; (iii) 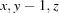; (iv) 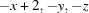; (v) 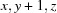.

**Table 3:** Experimental details

	(I)	(II)
Crystal data		
Chemical formula	C_19_H_20_N_2_O_2_	C_23_H_20_N_2_O_3_
*M*_r_	308.37	372.41
Crystal system, space group	Monoclinic, *P*2_1_	Triclinic, *P* 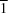
Temperature (K)	100	100
*a*, *b*, *c* (Å)	12.4525 (9), 5.7360 (4), 12.5896 (9)	9.2014 (5), 9.4543 (7), 21.6201 (14)
α, β, γ (°)	90, 116.081 (6), 90	98.563 (4), 93.416 (4), 98.354 (4)
*V* (Å^3^)	807.68 (11)	1833.7 (2)
*Z*	2	4
Radiation type	Mo *K*α	Mo *K*α
μ (mm^−1^)	0.08	0.09
Crystal size (mm)	0.28 × 0.05 × 0.01	0.10 × 0.06 × 0.06

Data collection		
Diffractometer	Rigaku Mercury CCD	Rigaku Mercury CCD
No. of measured, independent and observed [*I* > 2σ(*I*)] reflections	7830, 3498, 2259	24774, 8621, 6769
*R*_int_	0.107	0.038
(sin θ/λ)_max_ (Å^−1^)	0.649	0.668

Refinement		
*R*[*F* ^2^ > 2σ(*F* ^2^)], *wR*(*F* ^2^), *S*	0.081, 0.164, 1.12	0.046, 0.124, 1.03
No. of reflections	3498	8621
No. of parameters	213	513
No. of restraints	1	0
H-atom treatment	H atoms treated by a mixture of independent and constrained refinement	H atoms treated by a mixture of independent and constrained refinement
Δρ_max_, Δρ_min_ (e Å^−3^)	0.28, −0.24	0.62, −0.31

Computer programs: *CrystalClear* (Rigaku, 2012 ▸), *SHELXS97* (Sheldrick, 2008 ▸), *SHELXL2014* (Sheldrick, 2015 ▸), *ORTEP-3 for Windows* (Farrugia, 2012 ▸) and *publCIF* (Westrip, 2010 ▸).

## supplementary crystallographic information

### (I) 6-Isopropyl-3-(2-nitro-1-phenylethyl)-1H-indole Crystal data

**Table:**

C_19_H_20_N_2_O_2_	*F*(000) = 328
*M**_r_* = 308.37	*D*_x_ = 1.268 Mg m^−^^3^
Monoclinic, *P*2_1_	Mo *K*α radiation, λ = 0.71073 Å
*a* = 12.4525 (9) Å	Cell parameters from 6780 reflections
*b* = 5.7360 (4) Å	θ = 3.1–27.5°
*c* = 12.5896 (9) Å	µ = 0.08 mm^−^^1^
β = 116.081 (6)°	*T* = 100 K
*V* = 807.68 (11) Å^3^	Blade, light red
*Z* = 2	0.28 × 0.05 × 0.01 mm

### (I) 6-Isopropyl-3-(2-nitro-1-phenylethyl)-1H-indole Data collection

**Table:**

Rigaku Mercury CCD diffractometer	*R*_int_ = 0.107
ω scans	θ_max_ = 27.5°, θ_min_ = 3.1°
7830 measured reflections	*h* = −13→16
3498 independent reflections	*k* = −7→7
2259 reflections with *I* > 2σ(*I*)	*l* = −16→13

### (I) 6-Isopropyl-3-(2-nitro-1-phenylethyl)-1H-indole Refinement

**Table:**

Refinement on *F*^2^	1 restraint
Least-squares matrix: full	Hydrogen site location: mixed
*R*[*F*^2^ > 2σ(*F*^2^)] = 0.081	H atoms treated by a mixture of independent and constrained refinement
*wR*(*F*^2^) = 0.164	*w* = 1/[σ^2^(*F*_o_^2^) + (0.0357*P*)^2^ + 0.4188*P*] where *P* = (*F*_o_^2^ + 2*F*_c_^2^)/3
*S* = 1.12	(Δ/σ)_max_ < 0.001
3498 reflections	Δρ_max_ = 0.28 e Å^−^^3^
213 parameters	Δρ_min_ = −0.24 e Å^−^^3^

### (I) 6-Isopropyl-3-(2-nitro-1-phenylethyl)-1H-indole Special details

**Table:**

Geometry. All esds (except the esd in the dihedral angle between two l.s. planes) are estimated using the full covariance matrix. The cell esds are taken into account individually in the estimation of esds in distances, angles and torsion angles; correlations between esds in cell parameters are only used when they are defined by crystal symmetry. An approximate (isotropic) treatment of cell esds is used for estimating esds involving l.s. planes.

### (I) 6-Isopropyl-3-(2-nitro-1-phenylethyl)-1H-indole Fractional atomic coordinates and isotropic or equivalent isotropic displacement parameters (Å^2^)

**Table:**

	*x*	*y*	*z*	*U*_iso_*/*U*_eq_	
C1	0.5003 (5)	0.1427 (9)	0.3881 (4)	0.0226 (13)	
C2	0.3850 (5)	0.0617 (12)	0.3609 (4)	0.0254 (13)	
H2	0.3737	−0.0752	0.3972	0.031*	
C3	0.2883 (5)	0.1850 (11)	0.2804 (5)	0.0264 (13)	
C4	0.3092 (5)	0.3871 (10)	0.2291 (4)	0.0257 (14)	
H4	0.2422	0.4726	0.1747	0.031*	
C5	0.4222 (5)	0.4675 (10)	0.2538 (4)	0.0241 (13)	
H5	0.4329	0.6061	0.2183	0.029*	
C6	0.5208 (5)	0.3389 (10)	0.3329 (4)	0.0239 (12)	
C7	0.6491 (5)	0.3646 (10)	0.3796 (4)	0.0245 (13)	
C8	0.6977 (5)	0.1894 (10)	0.4586 (5)	0.0256 (13)	
H8	0.7811	0.1642	0.5038	0.031*	
C9	0.7126 (5)	0.5512 (11)	0.3434 (4)	0.0232 (12)	
H9	0.6848	0.7063	0.3577	0.028*	
C10	0.8468 (5)	0.5371 (11)	0.4194 (4)	0.0263 (13)	
H10A	0.8643	0.5484	0.5040	0.032*	
H10B	0.8771	0.3851	0.4067	0.032*	
C11	0.6842 (4)	0.5377 (10)	0.2123 (4)	0.0222 (12)	
C12	0.7189 (5)	0.3446 (10)	0.1683 (4)	0.0264 (13)	
H12	0.7558	0.2160	0.2187	0.032*	
C13	0.6998 (5)	0.3383 (11)	0.0510 (5)	0.0293 (14)	
H13	0.7245	0.2066	0.0216	0.035*	
C14	0.6451 (5)	0.5229 (11)	−0.0224 (5)	0.0288 (14)	
H14	0.6323	0.5190	−0.1025	0.035*	
C15	0.6086 (5)	0.7141 (11)	0.0200 (5)	0.0280 (13)	
H15	0.5692	0.8400	−0.0315	0.034*	
C16	0.6298 (5)	0.7225 (11)	0.1384 (5)	0.0258 (13)	
H16	0.6065	0.8558	0.1680	0.031*	
C17	0.1618 (5)	0.0955 (10)	0.2462 (5)	0.0294 (14)	
H17	0.1681	−0.0383	0.2995	0.035*	
C18	0.1043 (5)	0.0033 (12)	0.1182 (5)	0.0405 (17)	
H18A	0.1532	−0.1237	0.1106	0.061*	
H18B	0.0994	0.1298	0.0639	0.061*	
H18C	0.0238	−0.0547	0.0986	0.061*	
C19	0.0831 (6)	0.2786 (12)	0.2629 (6)	0.0420 (18)	
H19A	0.0047	0.2109	0.2449	0.063*	
H19B	0.0732	0.4100	0.2096	0.063*	
H19C	0.1205	0.3334	0.3450	0.063*	
N1	0.6094 (4)	0.0531 (9)	0.4643 (4)	0.0260 (11)	
N2	0.9084 (4)	0.7309 (9)	0.3883 (4)	0.0267 (11)	
H1	0.625 (5)	−0.056 (11)	0.514 (5)	0.032*	
O1	0.9829 (4)	0.6817 (8)	0.3538 (4)	0.0414 (11)	
O2	0.8825 (4)	0.9308 (7)	0.4015 (4)	0.0376 (11)	

### (I) 6-Isopropyl-3-(2-nitro-1-phenylethyl)-1H-indole Atomic displacement parameters (Å^2^)

**Table:**

	*U*^11^	*U*^22^	*U*^33^	*U*^12^	*U*^13^	*U*^23^
C1	0.035 (3)	0.016 (3)	0.020 (3)	0.006 (2)	0.015 (2)	0.000 (2)
C2	0.035 (3)	0.022 (3)	0.026 (3)	−0.003 (3)	0.020 (3)	−0.002 (3)
C3	0.033 (3)	0.026 (4)	0.023 (3)	−0.003 (3)	0.015 (2)	−0.005 (3)
C4	0.033 (3)	0.023 (4)	0.022 (3)	0.002 (3)	0.013 (2)	−0.002 (2)
C5	0.037 (3)	0.017 (3)	0.022 (3)	0.001 (3)	0.017 (3)	0.000 (2)
C6	0.033 (3)	0.019 (3)	0.023 (3)	0.002 (3)	0.016 (2)	0.000 (3)
C7	0.034 (3)	0.022 (3)	0.020 (3)	0.002 (3)	0.015 (2)	−0.002 (2)
C8	0.025 (3)	0.027 (3)	0.028 (3)	0.004 (3)	0.015 (2)	−0.002 (3)
C9	0.031 (3)	0.016 (3)	0.025 (3)	0.000 (3)	0.015 (2)	0.003 (3)
C10	0.032 (3)	0.023 (3)	0.028 (3)	0.000 (3)	0.016 (2)	0.003 (3)
C11	0.027 (3)	0.017 (3)	0.023 (3)	−0.003 (3)	0.013 (2)	−0.001 (3)
C12	0.035 (3)	0.019 (3)	0.024 (3)	−0.004 (3)	0.012 (2)	0.003 (3)
C13	0.040 (3)	0.023 (3)	0.029 (3)	−0.003 (3)	0.019 (3)	−0.004 (3)
C14	0.042 (4)	0.024 (3)	0.025 (3)	−0.007 (3)	0.019 (3)	−0.005 (3)
C15	0.033 (3)	0.024 (3)	0.027 (3)	−0.001 (3)	0.013 (2)	0.005 (3)
C16	0.032 (3)	0.021 (3)	0.028 (3)	0.000 (3)	0.016 (2)	−0.001 (3)
C17	0.034 (3)	0.026 (4)	0.029 (3)	−0.003 (3)	0.015 (3)	0.003 (3)
C18	0.039 (4)	0.038 (5)	0.044 (4)	−0.005 (3)	0.018 (3)	−0.005 (3)
C19	0.036 (4)	0.041 (5)	0.057 (4)	−0.001 (3)	0.027 (3)	−0.009 (3)
N1	0.033 (3)	0.024 (3)	0.026 (2)	0.007 (2)	0.017 (2)	0.006 (2)
N2	0.028 (3)	0.024 (3)	0.029 (3)	0.000 (2)	0.013 (2)	−0.004 (2)
O1	0.048 (3)	0.033 (3)	0.057 (3)	−0.002 (2)	0.036 (2)	−0.010 (2)
O2	0.042 (3)	0.018 (2)	0.057 (3)	0.002 (2)	0.025 (2)	−0.006 (2)

### (I) 6-Isopropyl-3-(2-nitro-1-phenylethyl)-1H-indole Geometric parameters (Å, º)

**Table:**

C1—N1	1.372 (7)	C11—C12	1.389 (8)
C1—C2	1.400 (7)	C12—C13	1.389 (7)
C1—C6	1.404 (7)	C12—H12	0.9500
C2—C3	1.380 (8)	C13—C14	1.373 (8)
C2—H2	0.9500	C13—H13	0.9500
C3—C4	1.406 (8)	C14—C15	1.381 (8)
C3—C17	1.527 (8)	C14—H14	0.9500
C4—C5	1.380 (7)	C15—C16	1.397 (7)
C4—H4	0.9500	C15—H15	0.9500
C5—C6	1.403 (7)	C16—H16	0.9500
C5—H5	0.9500	C17—C19	1.513 (8)
C6—C7	1.447 (7)	C17—C18	1.541 (8)
C7—C8	1.355 (8)	C17—H17	1.0000
C7—C9	1.516 (8)	C18—H18A	0.9800
C8—N1	1.377 (7)	C18—H18B	0.9800
C8—H8	0.9500	C18—H18C	0.9800
C9—C10	1.519 (7)	C19—H19A	0.9800
C9—C11	1.530 (7)	C19—H19B	0.9800
C9—H9	1.0000	C19—H19C	0.9800
C10—N2	1.497 (7)	N1—H1	0.84 (6)
C10—H10A	0.9900	N2—O1	1.217 (6)
C10—H10B	0.9900	N2—O2	1.222 (6)
C11—C16	1.376 (8)		
			
N1—C1—C2	129.8 (5)	C13—C12—C11	120.4 (5)
N1—C1—C6	107.9 (5)	C13—C12—H12	119.8
C2—C1—C6	122.3 (5)	C11—C12—H12	119.8
C3—C2—C1	118.7 (5)	C14—C13—C12	119.9 (6)
C3—C2—H2	120.7	C14—C13—H13	120.1
C1—C2—H2	120.7	C12—C13—H13	120.1
C2—C3—C4	118.8 (5)	C13—C14—C15	120.2 (5)
C2—C3—C17	119.6 (5)	C13—C14—H14	119.9
C4—C3—C17	121.6 (5)	C15—C14—H14	119.9
C5—C4—C3	123.2 (5)	C14—C15—C16	120.0 (5)
C5—C4—H4	118.4	C14—C15—H15	120.0
C3—C4—H4	118.4	C16—C15—H15	120.0
C4—C5—C6	118.2 (5)	C11—C16—C15	120.0 (5)
C4—C5—H5	120.9	C11—C16—H16	120.0
C6—C5—H5	120.9	C15—C16—H16	120.0
C5—C6—C1	118.7 (5)	C19—C17—C3	112.2 (5)
C5—C6—C7	134.5 (5)	C19—C17—C18	110.6 (5)
C1—C6—C7	106.8 (5)	C3—C17—C18	111.0 (4)
C8—C7—C6	106.3 (5)	C19—C17—H17	107.6
C8—C7—C9	128.4 (5)	C3—C17—H17	107.6
C6—C7—C9	125.4 (5)	C18—C17—H17	107.6
C7—C8—N1	110.5 (5)	C17—C18—H18A	109.5
C7—C8—H8	124.8	C17—C18—H18B	109.5
N1—C8—H8	124.8	H18A—C18—H18B	109.5
C7—C9—C10	110.3 (5)	C17—C18—H18C	109.5
C7—C9—C11	112.7 (5)	H18A—C18—H18C	109.5
C10—C9—C11	110.3 (4)	H18B—C18—H18C	109.5
C7—C9—H9	107.8	C17—C19—H19A	109.5
C10—C9—H9	107.8	C17—C19—H19B	109.5
C11—C9—H9	107.8	H19A—C19—H19B	109.5
N2—C10—C9	110.1 (4)	C17—C19—H19C	109.5
N2—C10—H10A	109.6	H19A—C19—H19C	109.5
C9—C10—H10A	109.6	H19B—C19—H19C	109.5
N2—C10—H10B	109.6	C1—N1—C8	108.6 (5)
C9—C10—H10B	109.6	C1—N1—H1	129 (4)
H10A—C10—H10B	108.2	C8—N1—H1	122 (4)
C16—C11—C12	119.5 (5)	O1—N2—O2	123.6 (5)
C16—C11—C9	120.1 (5)	O1—N2—C10	118.7 (5)
C12—C11—C9	120.3 (5)	O2—N2—C10	117.7 (5)
			
N1—C1—C2—C3	179.9 (5)	C7—C9—C10—N2	−176.5 (4)
C6—C1—C2—C3	−2.6 (8)	C11—C9—C10—N2	58.4 (6)
C1—C2—C3—C4	−0.2 (7)	C7—C9—C11—C16	118.9 (6)
C1—C2—C3—C17	177.9 (5)	C10—C9—C11—C16	−117.3 (6)
C2—C3—C4—C5	1.0 (8)	C7—C9—C11—C12	−64.5 (6)
C17—C3—C4—C5	−177.0 (5)	C10—C9—C11—C12	59.3 (7)
C3—C4—C5—C6	0.9 (7)	C16—C11—C12—C13	0.6 (8)
C4—C5—C6—C1	−3.5 (7)	C9—C11—C12—C13	−176.0 (5)
C4—C5—C6—C7	179.8 (5)	C11—C12—C13—C14	−0.8 (8)
N1—C1—C6—C5	−177.5 (5)	C12—C13—C14—C15	−0.2 (8)
C2—C1—C6—C5	4.5 (8)	C13—C14—C15—C16	1.4 (8)
N1—C1—C6—C7	0.0 (5)	C12—C11—C16—C15	0.6 (8)
C2—C1—C6—C7	−178.0 (5)	C9—C11—C16—C15	177.2 (5)
C5—C6—C7—C8	176.5 (6)	C14—C15—C16—C11	−1.6 (8)
C1—C6—C7—C8	−0.4 (6)	C2—C3—C17—C19	126.2 (6)
C5—C6—C7—C9	−4.3 (9)	C4—C3—C17—C19	−55.7 (7)
C1—C6—C7—C9	178.7 (5)	C2—C3—C17—C18	−109.4 (6)
C6—C7—C8—N1	0.7 (6)	C4—C3—C17—C18	68.6 (7)
C9—C7—C8—N1	−178.4 (5)	C2—C1—N1—C8	178.2 (5)
C8—C7—C9—C10	−6.4 (8)	C6—C1—N1—C8	0.4 (5)
C6—C7—C9—C10	174.6 (5)	C7—C8—N1—C1	−0.7 (6)
C8—C7—C9—C11	117.3 (6)	C9—C10—N2—O1	−120.8 (5)
C6—C7—C9—C11	−61.6 (7)	C9—C10—N2—O2	60.7 (6)

### (I) 6-Isopropyl-3-(2-nitro-1-phenylethyl)-1H-indole Hydrogen-bond geometry (Å, º)

Cg1, Cg2 and Cg3 are the centroids of the N1/C1/C6–C8, C1–C6 and C11–C16 rings, respectively.

**Table:**

*D*—H···*A*	*D*—H	H···*A*	*D*···*A*	*D*—H···*A*
N1—H1···*Cg*2^i^	0.84 (6)	2.64 (6)	3.386 (5)	148 (6)
C2—H2···*Cg*1^i^	0.95	2.63	3.468 (6)	147
C14—H14···*Cg*2^ii^	0.95	2.79	3.638 (6)	148
C15—H15···*Cg*3^ii^	0.95	2.87	3.551 (7)	129

Symmetry codes: (i) −*x*+1, *y*−1/2, −*z*+1; (ii) −*x*+1, *y*+1/2, −*z*.

### (II) 2-(4-Methoxyphenyl)-3-(2-nitro-1-phenylethyl)-1H-indole Crystal data

**Table:**

C_23_H_20_N_2_O_3_	*Z* = 4
*M**_r_* = 372.41	*F*(000) = 784
Triclinic, *P*1	*D*_x_ = 1.349 Mg m^−^^3^
*a* = 9.2014 (5) Å	Mo *K*α radiation, λ = 0.71073 Å
*b* = 9.4543 (7) Å	Cell parameters from 22268 reflections
*c* = 21.6201 (14) Å	θ = 2.2–27.5°
α = 98.563 (4)°	µ = 0.09 mm^−^^1^
β = 93.416 (4)°	*T* = 100 K
γ = 98.354 (4)°	Plate, colourless
*V* = 1833.7 (2) Å^3^	0.10 × 0.06 × 0.06 mm

### (II) 2-(4-Methoxyphenyl)-3-(2-nitro-1-phenylethyl)-1H-indole Data collection

**Table:**

Rigaku Mercury CCD diffractometer	*R*_int_ = 0.038
ω scans	θ_max_ = 28.4°, θ_min_ = 2.2°
24774 measured reflections	*h* = −11→11
8621 independent reflections	*k* = −12→12
6769 reflections with *I* > 2σ(*I*)	*l* = −28→28

### (II) 2-(4-Methoxyphenyl)-3-(2-nitro-1-phenylethyl)-1H-indole Refinement

**Table:**

Refinement on *F*^2^	Primary atom site location: structure-invariant direct methods
Least-squares matrix: full	Secondary atom site location: difference Fourier map
*R*[*F*^2^ > 2σ(*F*^2^)] = 0.046	Hydrogen site location: mixed
*wR*(*F*^2^) = 0.124	H atoms treated by a mixture of independent and constrained refinement
*S* = 1.03	*w* = 1/[σ^2^(*F*_o_^2^) + (0.0548*P*)^2^ + 0.8021*P*] where *P* = (*F*_o_^2^ + 2*F*_c_^2^)/3
8621 reflections	(Δ/σ)_max_ < 0.001
513 parameters	Δρ_max_ = 0.62 e Å^−^^3^
0 restraints	Δρ_min_ = −0.31 e Å^−^^3^

### (II) 2-(4-Methoxyphenyl)-3-(2-nitro-1-phenylethyl)-1H-indole Special details

**Table:**

Geometry. All esds (except the esd in the dihedral angle between two l.s. planes) are estimated using the full covariance matrix. The cell esds are taken into account individually in the estimation of esds in distances, angles and torsion angles; correlations between esds in cell parameters are only used when they are defined by crystal symmetry. An approximate (isotropic) treatment of cell esds is used for estimating esds involving l.s. planes.

### (II) 2-(4-Methoxyphenyl)-3-(2-nitro-1-phenylethyl)-1H-indole Fractional atomic coordinates and isotropic or equivalent isotropic displacement parameters (Å^2^)

**Table:**

	*x*	*y*	*z*	*U*_iso_*/*U*_eq_	
C1	0.49618 (16)	0.34164 (15)	0.06414 (6)	0.0170 (3)	
C2	0.38705 (17)	0.42054 (15)	0.08413 (7)	0.0189 (3)	
H2A	0.3201	0.4495	0.0551	0.023*	
C3	0.38042 (17)	0.45411 (16)	0.14638 (7)	0.0213 (3)	
H3A	0.3087	0.5094	0.1624	0.026*	
C4	0.47956 (18)	0.40736 (16)	0.18735 (7)	0.0222 (3)	
H4	0.4737	0.4329	0.2312	0.027*	
C5	0.58536 (17)	0.32607 (16)	0.16733 (7)	0.0199 (3)	
H5	0.6497	0.2950	0.1968	0.024*	
C6	0.59606 (16)	0.29082 (15)	0.10432 (7)	0.0171 (3)	
C7	0.68766 (16)	0.21018 (15)	0.06651 (7)	0.0177 (3)	
C8	0.64249 (16)	0.21756 (15)	0.00713 (7)	0.0173 (3)	
C9	0.81582 (17)	0.13561 (16)	0.08290 (7)	0.0217 (3)	
H9	0.8720	0.1249	0.0447	0.026*	
C10	0.92103 (18)	0.22377 (17)	0.13441 (8)	0.0245 (3)	
H10A	1.0027	0.1698	0.1428	0.029*	
H10B	0.8695	0.2389	0.1732	0.029*	
C11	0.77242 (16)	−0.01722 (16)	0.09801 (7)	0.0188 (3)	
C12	0.84562 (17)	−0.12686 (16)	0.07252 (7)	0.0217 (3)	
H12	0.9215	−0.1060	0.0458	0.026*	
C13	0.81037 (18)	−0.26753 (17)	0.08526 (8)	0.0254 (3)	
H13	0.8620	−0.3414	0.0672	0.030*	
C14	0.70126 (18)	−0.29942 (17)	0.12382 (7)	0.0244 (3)	
H14	0.6775	−0.3949	0.1329	0.029*	
C15	0.62638 (18)	−0.19130 (17)	0.14925 (7)	0.0243 (3)	
H15	0.5502	−0.2127	0.1758	0.029*	
C16	0.66181 (17)	−0.05090 (16)	0.13627 (7)	0.0214 (3)	
H16	0.6092	0.0225	0.1540	0.026*	
C17	0.70037 (16)	0.15610 (15)	−0.04944 (7)	0.0180 (3)	
C18	0.77075 (17)	0.24493 (16)	−0.08755 (7)	0.0212 (3)	
H18	0.7800	0.3471	−0.0764	0.025*	
C19	0.82656 (17)	0.18773 (16)	−0.14060 (7)	0.0214 (3)	
H19	0.8735	0.2487	−0.1671	0.026*	
C20	0.81369 (16)	0.03855 (16)	−0.15535 (7)	0.0190 (3)	
C21	0.74181 (17)	−0.05225 (16)	−0.11828 (7)	0.0207 (3)	
H21	0.7316	−0.1544	−0.1297	0.025*	
C22	0.68649 (17)	0.00643 (16)	−0.06567 (7)	0.0204 (3)	
H22	0.6379	−0.0547	−0.0396	0.025*	
C23	0.9661 (2)	0.06002 (19)	−0.23777 (7)	0.0297 (4)	
H23A	1.0146	−0.0009	−0.2682	0.045*	
H23B	1.0409	0.1233	−0.2079	0.045*	
H23C	0.9083	0.1192	−0.2599	0.045*	
N1	0.52777 (14)	0.29681 (13)	0.00553 (6)	0.0173 (2)	
H1	0.471 (2)	0.3091 (19)	−0.0271 (9)	0.021*	
N2	0.98391 (15)	0.36924 (15)	0.11821 (7)	0.0272 (3)	
O1	1.01892 (15)	0.37644 (15)	0.06681 (6)	0.0391 (3)	
O2	1.00019 (16)	0.47097 (14)	0.15869 (7)	0.0418 (3)	
O3	0.87109 (12)	−0.02940 (12)	−0.20477 (5)	0.0229 (2)	
C24	1.07078 (17)	0.27498 (15)	0.60018 (7)	0.0186 (3)	
C25	1.19481 (18)	0.23268 (16)	0.62786 (7)	0.0222 (3)	
H25	1.2510	0.1700	0.6046	0.027*	
C26	1.23137 (18)	0.28475 (17)	0.68904 (7)	0.0245 (3)	
H26	1.3142	0.2580	0.7102	0.029*	
C27	1.14771 (19)	0.37763 (17)	0.72123 (7)	0.0252 (3)	
H27	1.1756	0.4136	0.7643	0.030*	
C28	1.02574 (18)	0.42001 (17)	0.69332 (7)	0.0224 (3)	
H28	0.9715	0.4842	0.7169	0.027*	
C29	0.98380 (16)	0.36824 (15)	0.63114 (7)	0.0182 (3)	
C30	0.86833 (17)	0.38853 (16)	0.58743 (7)	0.0184 (3)	
C31	0.88891 (16)	0.30774 (15)	0.53304 (6)	0.0178 (3)	
C32	0.73865 (18)	0.46891 (17)	0.59473 (7)	0.0222 (3)	
H32	0.6627	0.4216	0.5600	0.027*	
C33	0.6680 (2)	0.45620 (18)	0.65431 (8)	0.0279 (4)	
H33A	0.5817	0.5077	0.6551	0.033*	
H33B	0.7389	0.5043	0.6901	0.033*	
C34	0.76883 (17)	0.62946 (16)	0.58830 (7)	0.0203 (3)	
C35	0.89706 (19)	0.72001 (17)	0.61460 (7)	0.0241 (3)	
H35	0.9719	0.6818	0.6360	0.029*	
C36	0.9164 (2)	0.86699 (18)	0.60972 (7)	0.0272 (4)	
H36	1.0045	0.9280	0.6278	0.033*	
C37	0.8086 (2)	0.92481 (18)	0.57886 (8)	0.0296 (4)	
H37	0.8214	1.0254	0.5767	0.035*	
C38	0.6826 (2)	0.83509 (19)	0.55131 (8)	0.0297 (4)	
H38	0.6091	0.8733	0.5291	0.036*	
C39	0.66297 (18)	0.68832 (18)	0.55610 (7)	0.0252 (3)	
H39	0.5757	0.6273	0.5370	0.030*	
C40	0.80143 (17)	0.28679 (16)	0.47391 (7)	0.0186 (3)	
C41	0.78144 (17)	0.40508 (16)	0.44448 (7)	0.0204 (3)	
H41	0.8257	0.4998	0.4637	0.024*	
C42	0.69952 (17)	0.38631 (17)	0.38876 (7)	0.0216 (3)	
H42	0.6880	0.4669	0.3684	0.026*	
C43	0.63290 (17)	0.24818 (17)	0.36192 (7)	0.0213 (3)	
C44	0.65272 (18)	0.12873 (17)	0.39006 (7)	0.0231 (3)	
H44	0.6082	0.0341	0.3709	0.028*	
C45	0.73698 (18)	0.14931 (16)	0.44560 (7)	0.0219 (3)	
H45	0.7516	0.0683	0.4651	0.026*	
C46	0.4670 (2)	0.1044 (2)	0.28258 (8)	0.0346 (4)	
H46A	0.4059	0.1135	0.2451	0.052*	
H46B	0.4035	0.0696	0.3138	0.052*	
H46C	0.5345	0.0355	0.2712	0.052*	
N3	1.01008 (14)	0.23898 (13)	0.54058 (6)	0.0186 (3)	
H3	1.050 (2)	0.193 (2)	0.5094 (9)	0.022*	
N4	0.61859 (17)	0.30122 (17)	0.66247 (8)	0.0351 (4)	
O4	0.55989 (17)	0.21888 (16)	0.61780 (8)	0.0474 (4)	
O5	0.6370 (2)	0.26938 (18)	0.71306 (8)	0.0575 (4)	
O6	0.54974 (13)	0.24217 (13)	0.30834 (5)	0.0278 (3)	

### (II) 2-(4-Methoxyphenyl)-3-(2-nitro-1-phenylethyl)-1H-indole Atomic displacement parameters (Å^2^)

**Table:**

	*U*^11^	*U*^22^	*U*^33^	*U*^12^	*U*^13^	*U*^23^
C1	0.0182 (7)	0.0134 (6)	0.0185 (6)	−0.0002 (5)	−0.0014 (5)	0.0033 (5)
C2	0.0184 (7)	0.0157 (7)	0.0230 (7)	0.0027 (6)	−0.0002 (6)	0.0054 (5)
C3	0.0219 (8)	0.0159 (7)	0.0263 (7)	0.0025 (6)	0.0044 (6)	0.0034 (6)
C4	0.0270 (8)	0.0195 (7)	0.0189 (7)	0.0001 (6)	0.0012 (6)	0.0029 (5)
C5	0.0226 (8)	0.0170 (7)	0.0197 (7)	0.0002 (6)	−0.0027 (6)	0.0056 (5)
C6	0.0180 (7)	0.0117 (6)	0.0209 (7)	−0.0004 (5)	−0.0022 (5)	0.0045 (5)
C7	0.0164 (7)	0.0136 (6)	0.0231 (7)	0.0004 (5)	−0.0009 (5)	0.0053 (5)
C8	0.0157 (7)	0.0131 (6)	0.0229 (7)	0.0013 (5)	0.0004 (5)	0.0041 (5)
C9	0.0194 (8)	0.0192 (7)	0.0276 (8)	0.0037 (6)	−0.0007 (6)	0.0078 (6)
C10	0.0229 (8)	0.0216 (8)	0.0295 (8)	0.0031 (6)	−0.0026 (6)	0.0082 (6)
C11	0.0180 (7)	0.0173 (7)	0.0208 (7)	0.0020 (6)	−0.0053 (5)	0.0059 (5)
C12	0.0199 (8)	0.0226 (7)	0.0228 (7)	0.0048 (6)	−0.0024 (6)	0.0043 (6)
C13	0.0255 (9)	0.0196 (7)	0.0296 (8)	0.0062 (6)	−0.0080 (6)	0.0006 (6)
C14	0.0272 (9)	0.0171 (7)	0.0270 (8)	−0.0014 (6)	−0.0116 (6)	0.0074 (6)
C15	0.0236 (8)	0.0250 (8)	0.0233 (7)	−0.0018 (6)	−0.0039 (6)	0.0084 (6)
C16	0.0205 (8)	0.0202 (7)	0.0234 (7)	0.0048 (6)	−0.0029 (6)	0.0036 (6)
C17	0.0159 (7)	0.0179 (7)	0.0200 (7)	0.0030 (6)	−0.0021 (5)	0.0037 (5)
C18	0.0227 (8)	0.0162 (7)	0.0260 (7)	0.0057 (6)	0.0007 (6)	0.0053 (6)
C19	0.0217 (8)	0.0198 (7)	0.0244 (7)	0.0038 (6)	0.0022 (6)	0.0083 (6)
C20	0.0156 (7)	0.0234 (7)	0.0179 (7)	0.0043 (6)	−0.0022 (5)	0.0028 (5)
C21	0.0205 (8)	0.0157 (7)	0.0244 (7)	0.0007 (6)	−0.0011 (6)	0.0012 (5)
C22	0.0198 (8)	0.0185 (7)	0.0223 (7)	0.0000 (6)	0.0003 (6)	0.0045 (5)
C23	0.0347 (10)	0.0337 (9)	0.0195 (7)	−0.0004 (7)	0.0050 (7)	0.0048 (6)
N1	0.0175 (6)	0.0176 (6)	0.0174 (6)	0.0048 (5)	−0.0016 (5)	0.0041 (4)
N2	0.0180 (7)	0.0245 (7)	0.0394 (8)	0.0016 (5)	−0.0014 (6)	0.0094 (6)
O1	0.0357 (8)	0.0405 (8)	0.0374 (7)	−0.0061 (6)	0.0004 (6)	0.0074 (6)
O2	0.0470 (9)	0.0270 (7)	0.0482 (8)	0.0013 (6)	0.0062 (7)	−0.0006 (6)
O3	0.0264 (6)	0.0230 (5)	0.0185 (5)	0.0029 (5)	0.0020 (4)	0.0021 (4)
C24	0.0212 (8)	0.0158 (7)	0.0178 (6)	−0.0010 (6)	−0.0010 (5)	0.0048 (5)
C25	0.0234 (8)	0.0185 (7)	0.0247 (7)	0.0028 (6)	−0.0012 (6)	0.0055 (6)
C26	0.0231 (8)	0.0250 (8)	0.0252 (8)	0.0001 (6)	−0.0065 (6)	0.0105 (6)
C27	0.0299 (9)	0.0271 (8)	0.0168 (7)	−0.0023 (7)	−0.0030 (6)	0.0062 (6)
C28	0.0258 (8)	0.0230 (7)	0.0173 (7)	0.0004 (6)	0.0010 (6)	0.0038 (5)
C29	0.0195 (7)	0.0179 (7)	0.0172 (6)	0.0004 (6)	0.0002 (5)	0.0056 (5)
C30	0.0189 (7)	0.0183 (7)	0.0179 (6)	0.0007 (6)	0.0005 (5)	0.0048 (5)
C31	0.0191 (7)	0.0163 (7)	0.0180 (7)	0.0001 (6)	−0.0011 (5)	0.0057 (5)
C32	0.0220 (8)	0.0213 (7)	0.0237 (7)	0.0026 (6)	0.0020 (6)	0.0055 (6)
C33	0.0297 (9)	0.0270 (8)	0.0284 (8)	0.0050 (7)	0.0059 (7)	0.0073 (6)
C34	0.0227 (8)	0.0210 (7)	0.0184 (7)	0.0051 (6)	0.0055 (6)	0.0042 (5)
C35	0.0278 (9)	0.0262 (8)	0.0183 (7)	0.0036 (7)	0.0014 (6)	0.0040 (6)
C36	0.0349 (10)	0.0245 (8)	0.0197 (7)	−0.0024 (7)	0.0072 (6)	0.0002 (6)
C37	0.0421 (11)	0.0208 (8)	0.0288 (8)	0.0062 (7)	0.0147 (7)	0.0077 (6)
C38	0.0317 (9)	0.0304 (9)	0.0329 (9)	0.0118 (7)	0.0099 (7)	0.0149 (7)
C39	0.0235 (8)	0.0276 (8)	0.0262 (8)	0.0049 (7)	0.0043 (6)	0.0079 (6)
C40	0.0181 (7)	0.0210 (7)	0.0164 (6)	0.0013 (6)	−0.0002 (5)	0.0049 (5)
C41	0.0214 (8)	0.0192 (7)	0.0195 (7)	−0.0007 (6)	−0.0001 (6)	0.0044 (5)
C42	0.0225 (8)	0.0228 (7)	0.0204 (7)	0.0017 (6)	−0.0011 (6)	0.0092 (6)
C43	0.0199 (8)	0.0271 (8)	0.0162 (6)	0.0008 (6)	−0.0024 (5)	0.0056 (6)
C44	0.0261 (8)	0.0209 (7)	0.0204 (7)	0.0001 (6)	−0.0026 (6)	0.0027 (6)
C45	0.0258 (8)	0.0200 (7)	0.0197 (7)	0.0024 (6)	−0.0023 (6)	0.0059 (6)
C46	0.0381 (11)	0.0361 (10)	0.0236 (8)	−0.0102 (8)	−0.0110 (7)	0.0062 (7)
N3	0.0206 (7)	0.0196 (6)	0.0152 (6)	0.0030 (5)	−0.0014 (5)	0.0028 (5)
N4	0.0287 (8)	0.0320 (8)	0.0469 (9)	0.0046 (7)	0.0123 (7)	0.0104 (7)
O4	0.0411 (8)	0.0394 (8)	0.0608 (10)	−0.0023 (7)	0.0144 (7)	0.0094 (7)
O5	0.0674 (11)	0.0575 (10)	0.0579 (10)	0.0112 (8)	0.0167 (8)	0.0370 (8)
O6	0.0310 (7)	0.0303 (6)	0.0194 (5)	−0.0034 (5)	−0.0103 (5)	0.0078 (4)

### (II) 2-(4-Methoxyphenyl)-3-(2-nitro-1-phenylethyl)-1H-indole Geometric parameters (Å, º)

**Table:**

C1—N1	1.3377 (18)	C24—N3	1.3478 (18)
C1—C2	1.387 (2)	C24—C25	1.394 (2)
C1—C6	1.410 (2)	C24—C29	1.400 (2)
C2—C3	1.343 (2)	C25—C26	1.345 (2)
C2—H2A	0.9500	C25—H25	0.9500
C3—C4	1.396 (2)	C26—C27	1.388 (2)
C3—H3A	0.9500	C26—H26	0.9500
C4—C5	1.376 (2)	C27—C28	1.381 (2)
C4—H4	0.9500	C27—H27	0.9500
C5—C6	1.366 (2)	C28—C29	1.372 (2)
C5—H5	0.9500	C28—H28	0.9500
C6—C7	1.429 (2)	C29—C30	1.432 (2)
C7—C8	1.341 (2)	C30—C31	1.343 (2)
C7—C9	1.510 (2)	C30—C32	1.509 (2)
C8—N1	1.3820 (19)	C31—N3	1.3830 (19)
C8—C17	1.440 (2)	C31—C40	1.441 (2)
C9—C10	1.494 (2)	C32—C33	1.490 (2)
C9—C11	1.530 (2)	C32—C34	1.531 (2)
C9—H9	1.0000	C32—H32	1.0000
C10—N2	1.512 (2)	C33—N4	1.509 (2)
C10—H10A	0.9900	C33—H33A	0.9900
C10—H10B	0.9900	C33—H33B	0.9900
C11—C16	1.381 (2)	C34—C35	1.389 (2)
C11—C12	1.382 (2)	C34—C39	1.391 (2)
C12—C13	1.394 (2)	C35—C36	1.396 (2)
C12—H12	0.9500	C35—H35	0.9500
C13—C14	1.371 (2)	C36—C37	1.383 (3)
C13—H13	0.9500	C36—H36	0.9500
C14—C15	1.380 (2)	C37—C38	1.379 (3)
C14—H14	0.9500	C37—H37	0.9500
C15—C16	1.394 (2)	C38—C39	1.393 (2)
C15—H15	0.9500	C38—H38	0.9500
C16—H16	0.9500	C39—H39	0.9500
C17—C18	1.384 (2)	C40—C45	1.383 (2)
C17—C22	1.391 (2)	C40—C41	1.395 (2)
C18—C19	1.356 (2)	C41—C42	1.357 (2)
C18—H18	0.9500	C41—H41	0.9500
C19—C20	1.385 (2)	C42—C43	1.385 (2)
C19—H19	0.9500	C42—H42	0.9500
C20—O3	1.3398 (17)	C43—O6	1.3389 (17)
C20—C21	1.386 (2)	C43—C44	1.389 (2)
C21—C22	1.351 (2)	C44—C45	1.363 (2)
C21—H21	0.9500	C44—H44	0.9500
C22—H22	0.9500	C45—H45	0.9500
C23—O3	1.4240 (19)	C46—O6	1.425 (2)
C23—H23A	0.9800	C46—H46A	0.9800
C23—H23B	0.9800	C46—H46B	0.9800
C23—H23C	0.9800	C46—H46C	0.9800
N1—H1	0.886 (19)	N3—H3	0.875 (18)
N2—O1	1.1841 (19)	N4—O5	1.186 (2)
N2—O2	1.1856 (19)	N4—O4	1.193 (2)
			
N1—C1—C2	128.86 (14)	N3—C24—C25	129.20 (14)
N1—C1—C6	106.46 (13)	N3—C24—C29	105.99 (13)
C2—C1—C6	124.68 (13)	C25—C24—C29	124.81 (14)
C3—C2—C1	116.96 (14)	C26—C25—C24	116.94 (15)
C3—C2—H2A	121.5	C26—C25—H25	121.5
C1—C2—H2A	121.5	C24—C25—H25	121.5
C2—C3—C4	119.63 (14)	C25—C26—C27	119.82 (15)
C2—C3—H3A	120.2	C25—C26—H26	120.1
C4—C3—H3A	120.2	C27—C26—H26	120.1
C5—C4—C3	123.24 (14)	C28—C27—C26	122.88 (14)
C5—C4—H4	118.4	C28—C27—H27	118.6
C3—C4—H4	118.4	C26—C27—H27	118.6
C6—C5—C4	118.74 (14)	C29—C28—C27	119.17 (15)
C6—C5—H5	120.6	C29—C28—H28	120.4
C4—C5—H5	120.6	C27—C28—H28	120.4
C5—C6—C1	116.72 (14)	C28—C29—C24	116.38 (14)
C5—C6—C7	134.97 (14)	C28—C29—C30	134.77 (14)
C1—C6—C7	108.31 (12)	C24—C29—C30	108.84 (12)
C8—C7—C6	105.06 (13)	C31—C30—C29	105.15 (13)
C8—C7—C9	122.53 (13)	C31—C30—C32	122.19 (14)
C6—C7—C9	132.32 (13)	C29—C30—C32	132.51 (13)
C7—C8—N1	110.65 (13)	C30—C31—N3	110.11 (13)
C7—C8—C17	127.68 (14)	C30—C31—C40	128.18 (14)
N1—C8—C17	121.66 (13)	N3—C31—C40	121.70 (13)
C10—C9—C7	112.94 (13)	C33—C32—C30	113.41 (13)
C10—C9—C11	109.64 (12)	C33—C32—C34	108.23 (12)
C7—C9—C11	114.72 (12)	C30—C32—C34	115.66 (13)
C10—C9—H9	106.3	C33—C32—H32	106.3
C7—C9—H9	106.3	C30—C32—H32	106.3
C11—C9—H9	106.3	C34—C32—H32	106.3
C9—C10—N2	112.11 (13)	C32—C33—N4	113.00 (14)
C9—C10—H10A	109.2	C32—C33—H33A	109.0
N2—C10—H10A	109.2	N4—C33—H33A	109.0
C9—C10—H10B	109.2	C32—C33—H33B	109.0
N2—C10—H10B	109.2	N4—C33—H33B	109.0
H10A—C10—H10B	107.9	H33A—C33—H33B	107.8
C16—C11—C12	118.10 (14)	C35—C34—C39	118.41 (14)
C16—C11—C9	122.57 (13)	C35—C34—C32	122.49 (14)
C12—C11—C9	119.33 (14)	C39—C34—C32	119.08 (14)
C11—C12—C13	121.32 (15)	C34—C35—C36	120.23 (16)
C11—C12—H12	119.3	C34—C35—H35	119.9
C13—C12—H12	119.3	C36—C35—H35	119.9
C14—C13—C12	120.07 (15)	C37—C36—C35	120.76 (16)
C14—C13—H13	120.0	C37—C36—H36	119.6
C12—C13—H13	120.0	C35—C36—H36	119.6
C13—C14—C15	119.29 (14)	C38—C37—C36	119.40 (15)
C13—C14—H14	120.4	C38—C37—H37	120.3
C15—C14—H14	120.4	C36—C37—H37	120.3
C14—C15—C16	120.43 (15)	C37—C38—C39	119.95 (16)
C14—C15—H15	119.8	C37—C38—H38	120.0
C16—C15—H15	119.8	C39—C38—H38	120.0
C11—C16—C15	120.79 (14)	C34—C39—C38	121.22 (16)
C11—C16—H16	119.6	C34—C39—H39	119.4
C15—C16—H16	119.6	C38—C39—H39	119.4
C18—C17—C22	119.76 (13)	C45—C40—C41	119.30 (14)
C18—C17—C8	120.44 (13)	C45—C40—C31	120.40 (13)
C22—C17—C8	119.80 (13)	C41—C40—C31	120.31 (13)
C19—C18—C17	120.71 (14)	C42—C41—C40	120.70 (14)
C19—C18—H18	119.6	C42—C41—H41	119.7
C17—C18—H18	119.6	C40—C41—H41	119.7
C18—C19—C20	118.60 (14)	C41—C42—C43	119.13 (14)
C18—C19—H19	120.7	C41—C42—H42	120.4
C20—C19—H19	120.7	C43—C42—H42	120.4
O3—C20—C19	123.68 (14)	O6—C43—C42	114.42 (13)
O3—C20—C21	114.75 (13)	O6—C43—C44	124.49 (14)
C19—C20—C21	121.55 (13)	C42—C43—C44	121.09 (14)
C22—C21—C20	119.10 (14)	C45—C44—C43	118.99 (14)
C22—C21—H21	120.4	C45—C44—H44	120.5
C20—C21—H21	120.4	C43—C44—H44	120.5
C21—C22—C17	120.25 (14)	C44—C45—C40	120.77 (14)
C21—C22—H22	119.9	C44—C45—H45	119.6
C17—C22—H22	119.9	C40—C45—H45	119.6
O3—C23—H23A	109.5	O6—C46—H46A	109.5
O3—C23—H23B	109.5	O6—C46—H46B	109.5
H23A—C23—H23B	109.5	H46A—C46—H46B	109.5
O3—C23—H23C	109.5	O6—C46—H46C	109.5
H23A—C23—H23C	109.5	H46A—C46—H46C	109.5
H23B—C23—H23C	109.5	H46B—C46—H46C	109.5
C1—N1—C8	109.50 (12)	C24—N3—C31	109.90 (12)
C1—N1—H1	120.6 (11)	C24—N3—H3	125.5 (12)
C8—N1—H1	129.3 (11)	C31—N3—H3	123.7 (12)
O1—N2—O2	122.88 (15)	O5—N4—O4	123.94 (18)
O1—N2—C10	119.27 (14)	O5—N4—C33	118.26 (17)
O2—N2—C10	117.80 (15)	O4—N4—C33	117.78 (16)
C20—O3—C23	116.20 (12)	C43—O6—C46	116.24 (13)
			
N1—C1—C2—C3	−178.82 (14)	N3—C24—C25—C26	−179.75 (15)
C6—C1—C2—C3	2.2 (2)	C29—C24—C25—C26	0.8 (2)
C1—C2—C3—C4	−1.0 (2)	C24—C25—C26—C27	−0.8 (2)
C2—C3—C4—C5	−0.5 (2)	C25—C26—C27—C28	0.3 (2)
C3—C4—C5—C6	1.0 (2)	C26—C27—C28—C29	0.3 (2)
C4—C5—C6—C1	0.1 (2)	C27—C28—C29—C24	−0.4 (2)
C4—C5—C6—C7	−179.51 (15)	C27—C28—C29—C30	−179.21 (16)
N1—C1—C6—C5	179.10 (13)	N3—C24—C29—C28	−179.75 (13)
C2—C1—C6—C5	−1.8 (2)	C25—C24—C29—C28	−0.2 (2)
N1—C1—C6—C7	−1.20 (16)	N3—C24—C29—C30	−0.62 (16)
C2—C1—C6—C7	177.94 (14)	C25—C24—C29—C30	178.93 (14)
C5—C6—C7—C8	−179.37 (16)	C28—C29—C30—C31	179.30 (17)
C1—C6—C7—C8	1.01 (16)	C24—C29—C30—C31	0.40 (16)
C5—C6—C7—C9	−2.8 (3)	C28—C29—C30—C32	−5.2 (3)
C1—C6—C7—C9	177.56 (14)	C24—C29—C30—C32	175.88 (15)
C6—C7—C8—N1	−0.45 (16)	C29—C30—C31—N3	−0.02 (16)
C9—C7—C8—N1	−177.42 (12)	C32—C30—C31—N3	−176.09 (13)
C6—C7—C8—C17	178.90 (14)	C29—C30—C31—C40	178.55 (14)
C9—C7—C8—C17	1.9 (2)	C32—C30—C31—C40	2.5 (2)
C8—C7—C9—C10	133.17 (15)	C31—C30—C32—C33	136.46 (16)
C6—C7—C9—C10	−42.9 (2)	C29—C30—C32—C33	−38.4 (2)
C8—C7—C9—C11	−100.19 (17)	C31—C30—C32—C34	−97.65 (17)
C6—C7—C9—C11	83.76 (19)	C29—C30—C32—C34	87.50 (19)
C7—C9—C10—N2	−58.42 (17)	C30—C32—C33—N4	−56.24 (19)
C11—C9—C10—N2	172.28 (13)	C34—C32—C33—N4	174.02 (14)
C10—C9—C11—C16	83.64 (18)	C33—C32—C34—C35	86.90 (18)
C7—C9—C11—C16	−44.7 (2)	C30—C32—C34—C35	−41.6 (2)
C10—C9—C11—C12	−96.58 (17)	C33—C32—C34—C39	−91.71 (17)
C7—C9—C11—C12	135.11 (15)	C30—C32—C34—C39	139.81 (14)
C16—C11—C12—C13	−0.6 (2)	C39—C34—C35—C36	1.4 (2)
C9—C11—C12—C13	179.65 (14)	C32—C34—C35—C36	−177.20 (13)
C11—C12—C13—C14	−0.1 (2)	C34—C35—C36—C37	0.1 (2)
C12—C13—C14—C15	0.6 (2)	C35—C36—C37—C38	−1.7 (2)
C13—C14—C15—C16	−0.4 (2)	C36—C37—C38—C39	1.6 (2)
C12—C11—C16—C15	0.7 (2)	C35—C34—C39—C38	−1.4 (2)
C9—C11—C16—C15	−179.52 (14)	C32—C34—C39—C38	177.22 (14)
C14—C15—C16—C11	−0.2 (2)	C37—C38—C39—C34	−0.1 (2)
C7—C8—C17—C18	−114.00 (18)	C30—C31—C40—C45	−121.49 (18)
N1—C8—C17—C18	65.3 (2)	N3—C31—C40—C45	56.9 (2)
C7—C8—C17—C22	65.5 (2)	C30—C31—C40—C41	58.8 (2)
N1—C8—C17—C22	−115.22 (16)	N3—C31—C40—C41	−122.77 (16)
C22—C17—C18—C19	−0.1 (2)	C45—C40—C41—C42	0.2 (2)
C8—C17—C18—C19	179.44 (14)	C31—C40—C41—C42	179.86 (14)
C17—C18—C19—C20	−1.0 (2)	C40—C41—C42—C43	1.4 (2)
C18—C19—C20—O3	−176.55 (14)	C41—C42—C43—O6	177.38 (14)
C18—C19—C20—C21	1.9 (2)	C41—C42—C43—C44	−2.2 (2)
O3—C20—C21—C22	176.83 (14)	O6—C43—C44—C45	−178.21 (15)
C19—C20—C21—C22	−1.8 (2)	C42—C43—C44—C45	1.3 (2)
C20—C21—C22—C17	0.7 (2)	C43—C44—C45—C40	0.3 (2)
C18—C17—C22—C21	0.2 (2)	C41—C40—C45—C44	−1.0 (2)
C8—C17—C22—C21	−179.29 (14)	C31—C40—C45—C44	179.25 (15)
C2—C1—N1—C8	−178.16 (14)	C25—C24—N3—C31	−178.91 (14)
C6—C1—N1—C8	0.93 (16)	C29—C24—N3—C31	0.60 (16)
C7—C8—N1—C1	−0.30 (17)	C30—C31—N3—C24	−0.37 (17)
C17—C8—N1—C1	−179.70 (13)	C40—C31—N3—C24	−179.06 (13)
C9—C10—N2—O1	−41.3 (2)	C32—C33—N4—O5	139.49 (17)
C9—C10—N2—O2	141.02 (15)	C32—C33—N4—O4	−42.3 (2)
C19—C20—O3—C23	8.6 (2)	C42—C43—O6—C46	−173.68 (15)
C21—C20—O3—C23	−169.94 (13)	C44—C43—O6—C46	5.8 (2)

### (II) 2-(4-Methoxyphenyl)-3-(2-nitro-1-phenylethyl)-1H-indole Hydrogen-bond geometry (Å, º)

Cg1, Cg2, Cg3, Cg6, Cg7 and Cg8 are the centroids of the N1/C1/C6–C8, C1–C6, C11–C16, N3/C24/C29–C31, C24–C29 and C34–C39 rings, respectively.

**Table:**

*D*—H···*A*	*D*—H	H···*A*	*D*···*A*	*D*—H···*A*
N1—H1···*Cg*3^i^	0.886 (19)	2.640 (19)	3.3631 (15)	139.6 (15)
N3—H3···*Cg*8^ii^	0.875 (18)	2.582 (19)	3.3364 (15)	144.9 (16)
C14—H14···*Cg*2^iii^	0.95	2.58	3.4228 (18)	149
C21—H21···*Cg*2^i^	0.95	2.69	3.4133 (17)	134
C22—H22···*Cg*1^i^	0.95	2.68	3.4543 (17)	138
C23—H23*B*···*Cg*3^iv^	0.98	2.79	3.6739 (18)	150
C37—H37···*Cg*6^v^	0.95	2.86	3.7660 (18)	160
C41—H41···*Cg*6^ii^	0.95	2.70	3.3793 (17)	129
C42—H42···*Cg*7^ii^	0.95	2.67	3.3627 (17)	130

Symmetry codes: (i) −*x*+1, −*y*, −*z*; (ii) −*x*+2, −*y*+1, −*z*+1; (iii) *x*, *y*−1, *z*; (iv) −*x*+2, −*y*, −*z*; (v) *x*, *y*+1, *z*.
